# Grasping Hand Verbs: Oscillatory Beta and Alpha Correlates of Action-Word Processing

**DOI:** 10.1371/journal.pone.0108059

**Published:** 2014-09-23

**Authors:** Valentina Niccolai, Anne Klepp, Hannah Weissler, Nienke Hoogenboom, Alfons Schnitzler, Katja Biermann-Ruben

**Affiliations:** Institute for Clinical Neuroscience and Medical Psychology, Medical Faculty, Heinrich Heine University, Düsseldorf, Germany; Birkbeck, University of London, United Kingdom

## Abstract

The grounded cognition framework proposes that sensorimotor brain areas, which are typically involved in perception and action, also play a role in linguistic processing. We assessed oscillatory modulation during visual presentation of single verbs and localized cortical motor regions by means of isometric contraction of hand and foot muscles. Analogously to oscillatory activation patterns accompanying voluntary movements, we expected a somatotopically distributed suppression of beta and alpha frequencies in the motor cortex during processing of body-related action verbs. Magnetoencephalographic data were collected during presentation of verbs that express actions performed using the hands (H) or feet (F). Verbs denoting no bodily movement (N) were used as a control. Between 150 and 500 msec after visual word onset, beta rhythms were suppressed in H and F in comparison with N in the left hemisphere. Similarly, alpha oscillations showed left-lateralized power suppression in the H-N contrast, although at a later stage. The cortical oscillatory activity that typically occurs during voluntary movements is therefore found to somatotopically accompany the processing of body-related verbs. The combination of a localizer task with the oscillatory investigation applied to verb reading as in the present study provides further methodological possibilities of tracking language processing in the brain.

## Introduction

Two main theories make assumptions on how the brain processes language and concepts. The amodal approach proposes that all concepts are processed in an amodal unit, independently from their modality [1], [2]. Differently, grounded (or embodied) cognition theories postulate that perceptual-motor processes are crucial in concept representation [3]–[5]. In this context, it is assumed that body-related action words are handled by the same brain areas involved in the execution of the respective movements. Language processing would thus include cortico-cortical connections between the classical temporal (Wernicke’s area) and inferior frontal (Broca’s area) language regions and the motor system [5]. It has been proposed that mirror neurons [6] and Hebbian association mechanisms [5], [7], [8] implement the functional overlap between action comprehension and execution. A middle ground between the embodied and disembodied cognition hypotheses has also been suggested [9].

A number of functional magnetic resonance imaging (fMRI) studies have tested the grounded cognition hypothesis and, with a few exceptions [10], have demonstrated the recruitment of cortical premotor and primary motor regions for the processing of action words or sentences [11], [12], [13], [14], [15], [16]. Moreover, transcranial magnetic stimulation (TMS) of the hand and foot motor areas during the processing of effector-specific action verbs and sentences modulates reaction times and cortical excitability [17]–[19]. Recently, our research group showed by means of magnetoencephalography (MEG) somatotopic activation of motor areas accompanying the processing of visually presented single verbs [20]. These findings consistently point to a somatotopically organized engagement of cortical motor areas in the understanding of written and spoken action.

Although specific patterns of cortical oscillatory activation are known to accompany limb movement execution, observation [21], [22], [23], and motor imagery [24], [25], [26], [27], [28], the oscillatory correlates of action word processing have hardly been addressed [29], [30], [31]. Power suppression of beta frequency is typically elicited by the preparation and execution of movements [21], [23], [32], [33] and by the isometric contraction of different body muscles [34], [35]. Similarly, a decrease of the alpha rhythm is known to accompany movement execution as well as motor imagery [36], [37]. In line with the postulation of grounded cognition theory, it is conceivable that the processing of body-related verbs induces beta and alpha power suppression in motor cortical areas that are engaged in the respective action execution. A few studies have focused on oscillatory cortical motor correlates of action words. Testing whether motor activation in verb processing reflects motor imagery or semantic processing, van Elk et al. [31] found stronger mu (10–14 Hz) and beta power suppression starting about 200 msec after verb onset in motor areas while processing animal compared to human action sentences. Due to early onset and inverse correlation to N400 peak amplitudes, the authors concluded that this may be a sign of lexical-semantic integration. Generation of an unspecific verb associated to a series of acoustically presented single nouns was shown to be accompanied by power suppression in the 15–25 Hz beta range on the left premotor cortex [38]. In addition to this, when reading hand-action versus abstract sentences, a decrease of mu rhythm was observed on left and central frontal leads [39]. Listening to verbal stimuli (pseudowords) that had been previously associated with movements resulted in suppression of the mu rhythm over the centro-parietal region [40]. What remains to be assessed is the somatotopic distribution of oscillatory modulations in motor brain areas. This is the first study that combined a localizer task with the oscillatory investigation of single verb processing, in order to explicitly test the embodiment theory. Using MEG, we compared hand- and foot-related verbs to verbs that involve no body movement, to which we refer as abstract verbs. We expected body-related words to induce a stronger beta (15–25 Hz) and alpha (7–11 Hz) power suppression in the respective sensorimotor cortices compared to non-body-related actions. As hands/arms occasionally move during foot-related actions, we chose to contrast each body-related verb condition against abstract verbs instead of against each other to maximize the sensitivity of the contrast. To localize hand and foot representations of the motor cortex, subjects performed isometric contractions of hand and foot muscles in two separate measurements which were further analysed offline. The resulting corticomuscular coherence represents the functional connectivity between a contralateral effector muscle and the sensorimotor and, possibly, the premotor cortex [41].

Capitalizing on the high time resolution of electroencephalography (EEG), it was shown that lexico-semantic processing related to bodily action words activated the cortical motor area around 200 msec after the presentation of the visual stimulus [13], [42]. Similarly, spoken body-related verbs elicited preponderantly left-hemispheric event-related potential or field in the sensorimotor cortex between 140 and 200 msec after stimulus onset [43], [44]. Since grounded cognition theories propose that the sensorimotor activation contributing to language understanding should occur within the time frame of lexico-semantic processes [45], we expected oscillatory modulations to emerge at about 200 msec post-stimulus onset. To select stimulus material and to control for psycholinguistic parameters that may affect word processing, rating studies were performed in advance. Individuals who did not take part in the MEG study were asked to evaluate the verbs’ body-relatedness, familiarity, and imageability. Although the task applied in the present MEG study did not demand movement imagery, we additionally tested whether implicit imagery processes affected the oscillatory modulations related to lexico-semantic processes.

## Materials and Methods

### Participants

Fifteen university students (8 women, aged 22 years, SD  = 1.8), all monolingual German native speakers, took part in the MEG study. All participants were right-handed, with an average laterality quotient of 84.1% (SD  = 16.2%; Edinburgh Handedness Inventory, [46]), and right-footed (Lateral Preference Inventory, [40]). The subjects had normal or corrected-to-normal vision and none reported neurological or psychiatric disorders or made use of neuro-modulatory medications. Participants provided written informed consent prior to the MEG and received financial compensation for their participation. The study was in accordance with the Declaration of Helsinki and was approved by the local Ethics Committee of the Medical Faculty of the Heinrich Heine University, Düsseldorf (study number 3400).

### Materials

Stimuli consisted of German disyllabic infinitive verbs describing actions done with the upper extremities (hand, H), actions done with the lower extremities (foot, F), and actions in which no body part was involved (N). To find suitable stimuli, 339 verbs were used in a computerized rating study. In the first rating study, 30 monolingual German speakers (17 women, aged 29.7 years, SD  = 6.8) specified which body part they usually use to perform the action described by each verb. Possible answers were “hands/arms”, “feet/legs”, “the whole body uniformly”, “mouth/face”, “no body part” and “I don’t know”. Categories that were not part of the main experimental focus (“mouth/face”, “whole body”) were applied to prevent forced choices of inaccurate answers. To be included in the sets of H, F and N, verbs had to be rated as describing actions of the respective body part by at least 80% of the subjects. For F, ratings were often split between “feet/legs” and “whole body”, possibly due to locomotion verbs (e.g., *to run*) being rated as “whole body” by some participants, who focused on the body’s change of location rather than the movements of the lower extremity. Therefore, for the F category, verbs were also included if the sum of “feet/legs” and “whole body” answers reached the 80% threshold, as long as at least 40% of the ratings were “feet/legs”. The resulting 219 H, F and N verbs were subjected to a second computerized rating study (n = 30, 16 women, aged 28.8 years, SD  = 6.4) in which subjects had to assess familiarity and imageability on 4-point rating scales. Mean familiarity, imageability, word length and word frequency class [47] (http://wortschatz.uni-leipzig.de) were used to define suitably matched groups of stimuli, resulting in 48 verbs per condition. While familiarity did not differ between groups (ANOVA, *p* = .54), residual differences were found for the other parameters (ANOVA, all *p*<.01). More precisely, according to paired tests, N verbs were on average 0.8 letters longer than H (*t_94_* = 3.09, *p* = .003) and F (*t_94_* = 2.70, *p* = .008), less imageable than H (*t_94_* = 23.33, *p*<.001) and F (*t_94_* = 18.08, *p*<.001), and more frequent than H (*t_94_* = 4.59, *p*<.001) and F (*t_94_* = 2.79, *p* = .006). The conditions H and F showed no significant differences (all *p*>.13). Fifty percent of the H verbs were unilateral actions. To control for the influence of imageability, stimulus sets were further divided into high and low imageability by a median split. For the lexical decision task introduced below, 18 pronounceable non-existent words (pseudoverbs) were created by reassembling the first and second syllables of the stimulus verbs. To this end, all first and second syllable occurrences in the data set were counted. Frequencies of pseudoverb endings (for German, typically ‘-en’, ‘-ern’ or ‘-eln’) as well as the initial letters of the first and second syllables were chosen to broadly resemble the main data set in order to avoid introducing a processing bias. Another 18 verbs (6 for each condition) that were discarded during the matching procedure were used as fillers. A list of the stimuli and relative parameters values is presented in Table S1.

### Procedure

**Figure 1:**
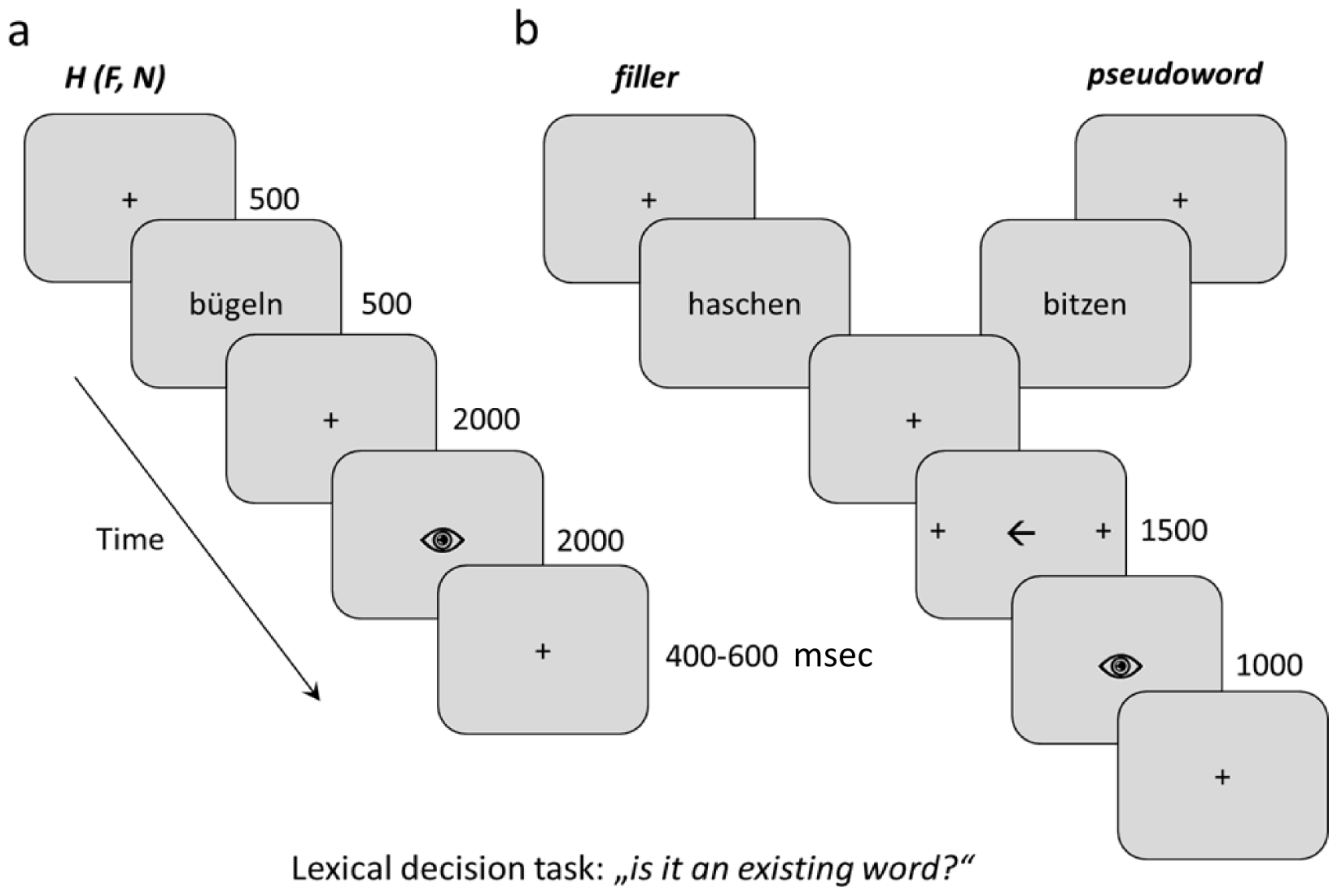
Experimental design. Trials which were not followed by a cue (a) were included in the analysis. The prompt to respond followed fillers and pseudowords (b).

Subjects removed all metallic objects and put on non-magnetic clothes prior to the MEG measurement to prevent recording artifacts. During the experimental session, participants were comfortably seated in a magnetically shielded room and viewed a screen at a distance of 83 cm. Black words were centrally presented against a light grey background and subtended a visual angle of 3.4° by .7° on average. Presentation software (version 14.9, Neurobehavioral Systems, Albany, California, USA) was used to display the stimuli. Each trial began with a central fixation cross displayed for 500 msec, followed by a word or a pseudoword that remained on the screen for 500 msec. The fixation cross then appeared again for 2 s and was followed by an eye symbol shown for 2 s, which indicated the time for blinking. A fixation cross with a jittered duration of between 400 and 600 msec ended the trial without perceivable intersection to the following trial (Fig. 1). Participants were instructed to identify whether the stimulus was an existing word. Responses had to be given in only 20% of all trials, namely with filler verbs and pseudoverbs. In these trials, responses were prompted by a central arrow pointing to one of two lateral fixation crosses at a distance of 6.8° to the centre of the arrow. This screen lasted for 1500 msec and was inserted after the fixation cross following verb presentation. Subjects had to switch their gaze from the centre to one of the lateral fixation crosses. In cases where a real verb (the filler) was presented, they had to look at the cross pointed to by the arrow. If it was a pseudoverb they had to look into the opposite direction. The arrow pseudo-randomly pointed to the right and left side. The response cue was followed by the eye symbol which was displayed for one second. To avoid alteration of brain oscillations due to eye movements, only stimuli that were not followed by a response cue were analysed. Importantly, the fillers were indistinguishable from the analysed stimuli. Stimuli were randomly presented and they were repeated in a second block. A break was inserted every 5 minutes. Overall, the measurement lasted about 40 minutes. A total of 16 stimuli (10 pseudowords/fillers and 6 action verbs) different from those of the main study were used in a practice session preceding the experiment. Horizontal eye movements were calibrated to improve the analysis of behavioural accuracy.

### Localizer task

To localize cortical sensorimotor areas corresponding to upper and lower extremities, subjects performed two isometric muscle contraction tasks. With their elbows resting on a table, they were required to bend their arms to about 30° and to simultaneously spread their fingers. Controlled by means of electromyographic (EMG) recordings, the contraction was limited to about 50% of the maximal strength. In the foot contraction condition, subjects raised their feet and toes upwards towards the body. Rest and contraction phases lasting one minute each were alternated twice. Participants were asked to fix their gaze straight ahead and to avoid eye movements during the contraction phase. Practice trials were performed before starting the measurement.

### Data Acquisition and Analysis

Neuromagnetic brain activity was continuously recorded with a 306-channel MEG system (Elekta Neuromag, Helsinki, Finland), including 204 orthogonal planar gradiometers and 102 magnetometers. A bipolar horizontal and vertical electrooculogram (EOG) was recorded for the offline detection of eye movements. Additionally, a bipolar EMG was recorded from the extensor digitorum communis (EDC) muscle of each forearm and from the tibialis anterior (TA) muscle of each leg. Four coils were attached to the subject’s head bilaterally on the forehead and behind the ears. The position of these coils, prominent anatomical landmarks (right and left preauricular points and nasion) and some additional points along the subject’s head were digitized (Polhemus Isotrak) to map functional MEG data to individual anatomy. MEG data were digitized at 1000 Hz, band-pass filtered from 0.03 to 330 Hz online, and stored on a computer hard disk. As for the analysis of behavioural data, the response accuracy of each subject was visually inspected on EOG traces using the Neuromag software package (Elekta Neuromag, Helsinki, Finland). MEG data were analysed with Matlab 2012a (Mathworks, Natick, MA, USA) and FieldTrip (http://fieldtrip.fcdonders.nl), a Matlab software toolbox for MEG and EEG analyses [48]. Data from 204 gradiometers were analysed.

#### Pre-processing of MEG data

Epochs from −500 to 1000 msec relative to verb onset were gathered from the continuous data. An additional 440 msec of data at the beginning and at the end of the epoch was included to avoid edge effects at low frequencies. Segments were created for the three conditions H, F, and N. For analyses of imageability effects, epochs from each condition were further segmented into high and low imageable sub-conditions. Data were filtered with a high-pass filter of 2 Hz and with band-stop filters at 49–51, 99–101, 149–151 Hz; a Butterworth IIR zero-phase forward and reverse filter was used. Segments containing artifacts related to blinks and to movements of the eyes, hands, and feet were removed by means of a semi-automatic algorithm. An average of 81 trials (±7 SD) in the H, 79 (±8 SD) in the F, and 79 (±9 SD) in the N condition passed artifacts rejection per subject. There was no significant difference among number of trials per condition (ANOVA, *F_(2,44)_* = .24, *p* = .78). Channels with bad signal were replaced with the average of their intact neighbours (nearest-neighbour approach; [20]). Independent component analysis (ICA; [49]) applied to the output of a principal component analysis was run to identify cardiac artifacts. Fifty components per subject were estimated and visually inspected. One to two components representing cardiac artifacts were eliminated from the data of each subject.

#### Channel selection

The localizer tasks described above analysed in terms of corticomuscular coherence provided channel selections for the analysis of the verbal paradigm. To this end, two data epochs of about 1 minute each during muscle contraction were used for coherence analysis. EOG artifacts were rejected. Both MEG and EMG data were notch-filtered at 50 Hz power-supply noise frequency. EMG data were additionally filtered using a high-pass Butterworth IIR zero-phase forward and reverse filter at 10 Hz and rectified. The data were then segmented in 1 s trials. Time-frequency representations (TFR) were calculated using a multitaper method based on discrete prolate spheroidal sequences (DPSS) tapers which created a spectral smoothing of ±5 Hz. Cross-spectra frequency and coherence were computed between MEG channels and each EMG channel. Grand-average maps were visually inspected and MEG sensors showing coherence to right and left hand and foot were selected for further analyses of the word paradigm.

#### Time-frequency analysis

TFR were calculated by means of a fast Fourier transform (FFT). An adaptive window including 5 cycles was shifted in steps of 50 msec from −500 to 1000 msec. Data were padded up to 3 s. A Hanning taper was applied to the epochs. Power was estimated between 5 and 39 Hz in steps of 2 Hz. A time-frequency analysis was separately applied to horizontal and vertical planar gradiometers. The pairs of planar gradiometers were then combined and trials were normalised with respect to the baseline, which included pre-stimulus data between −500 and −100 msec. Importantly, power representations in the baselines did not significantly differ between the H and N or between the F and N condition (all *p*>.2), according to the cluster-based randomization test described in the ‘Statistical analysis of MEG data’ section. To avoid an overlap in the frequency resolution between beta and alpha oscillations, the alpha rhythm was defined as being between 7 and 11 Hz while beta rhythm was specified as 15 to 25 Hz. Time-frequency analysis resulted in a resolution of 3–5 Hz for beta and 1.4–2.2 Hz for alpha.

#### Statistical analysis of MEG data

**Figure 2:**
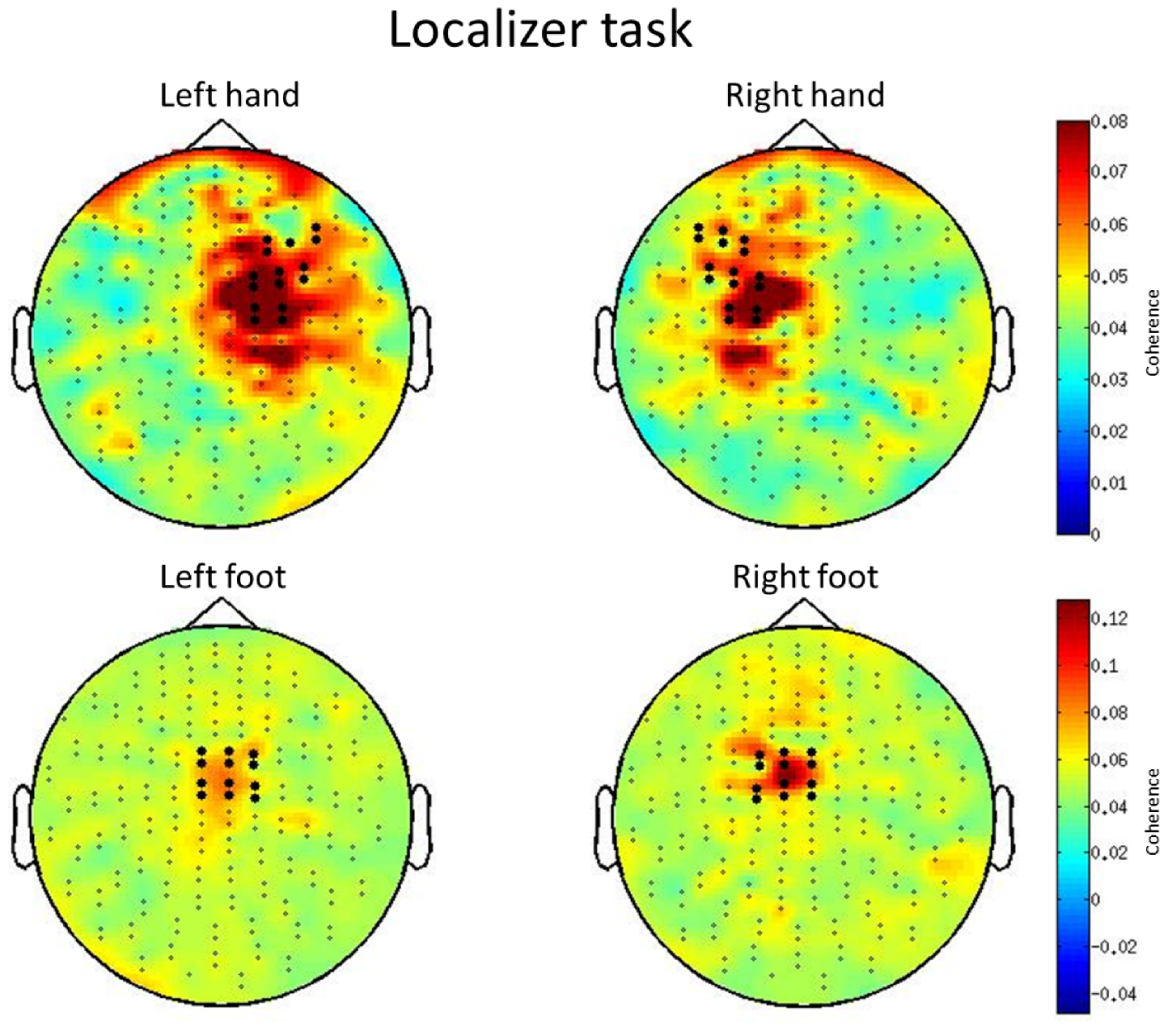
Grand-average of corticomuscular coherence in the beta (15–25 Hz) range related to hands (top) and to feet (bottom) isometric contraction across 15 subjects. Bold points represent gradiometer pairs selected for frequency analysis in the word paradigm.

Statistical analysis of the MEG data consisted of a two-step procedure that effectively corrects for multiple comparisons and that has been applied previously [50]–[52]. First, the power difference between condition H and control condition N was calculated by means of t-values. T-values were calculated for each sensor, frequency bin and time point of each subject. In a second step, a cluster-based non-parametric randomization approach was used to test significance at group level [53]. The group analysis was run based on the average of the selected sensors (see *Channel selection*) and on a time-window of interest between 150 and 500 msec after word onset. According to the null hypothesis, the difference between H and N should not significantly differ from zero, that is, *t*-values should be replaceable by zero. Thus, resulting *t*-values of each subject and values from a pseudo-dataset consisting of zeros went through a random partition which involved a shuffling of data between the two datasets. Time-frequency maps exceeding an a priori threshold (uncorrected *p*<.05) were combined into clusters. A cluster containing the summed *t*-values was used to calculate a cluster-level test statistic. The random partition was repeated 1000 times, every time resulting in a cluster-level test statistic calculated for the re-shuffled data. The subsequent histogram of the summed *t*-values constituted the cluster-based randomization test. The proportion of test statistics which were larger or smaller, respectively than the calculated statistic based on the observed original H-N contrast constituted the *p*-value. In cases where the *p*-value was smaller than an alpha-level of 0.05, we concluded that data in the two conditions H and N were significantly different. Given the well-known left-hemispheric specialization for language, this two-step statistical procedure was applied separately to the averages of the selected sensors of the left and right hemisphere for the H-N contrast. Due to the central location and overlap, the sensor selection for the F-N contrast included those related to the right and to the left foot taken together (Fig. 2), thus resulting in a total of 8 channels pairs, not averaged.

Using a similar statistical procedure, we tested whether the lexico-semantic oscillatory modulations were confounded by imageability effects. To test the main effects of imageability, we calculated the mean power across the H, F, and N condition (high vs. low imageability), thus resulting in two datasets each including all conditions, and we compared high versus low imagery subconditions on the selected hand and foot motor areas by means of the cluster randomization approach described above. To test a possible interaction between imageability and lexico-semantic effects, we calculated the differences between the H and N as well as between the F and N condition (high vs. low imageability), and we compared high versus low imageable datasets on the selected concordant hand and foot motor areas.

## Results

### Behavioural results

Participants successfully performed the task with an average accuracy of 89% (SD  = 6.2%). This indicates that they were paying attention to the presented words. All subjects responded to each prompt with the exception of one subject, who failed to respond to 12% of the cued trials.

### MEG results

#### Localizer task

Corticomuscular coherence in the 15–25 Hz beta-range during isometric contraction of hands showed a fronto-parietal distribution on the hemisphere contralateral to the contracted hand (Fig. 2). Contraction of feet activated a centrally located motor area and showed only slight lateralization. Eight hand channel pairs per hemisphere (bold points in Fig. 2) were selected for analysis of the H-N contrast in the word paradigm. As the two groups of foot-related channels largely overlapped, the sum of them (8 channel pairs) was selected for the analysis of the F-N contrast.

#### Word paradigm

**Figure 3:**
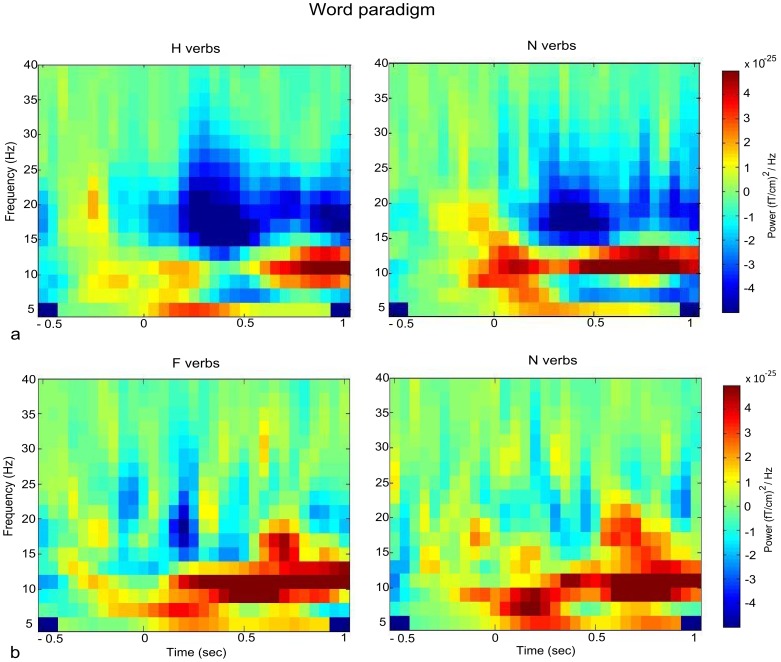
Grand-average of frequency spectra. a) Grand-average of the H (left) and the N (right) condition on the average of the left-hemispheric hand-related sensors selected with the localizer task. b) Grand-average of the F (left) and the N (right) condition on the average of three foot-related sensors showing a significant effect.

**Figure 4:**
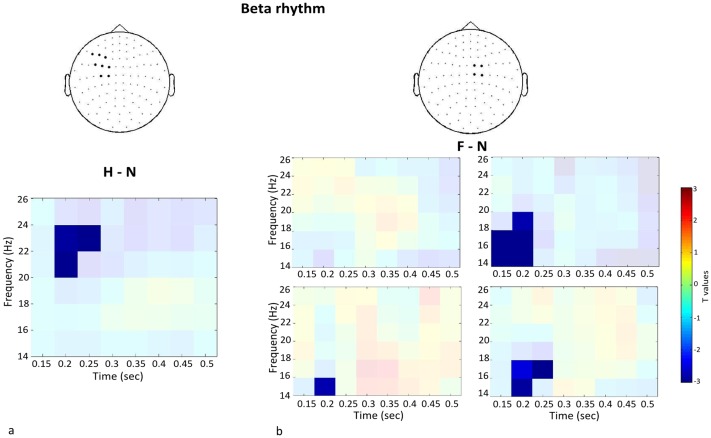
Statistical outcomes for the beta rhythm. a) T-values on a time-frequency map related to the H-N contrast, showing a significant cluster (saturated colours) on the average of the selected left-hemispheric hand-related sensors. b) Time-frequency maps of the F-N contrast showing a significant cluster on three foot-related sensors.

**Figure 5:**
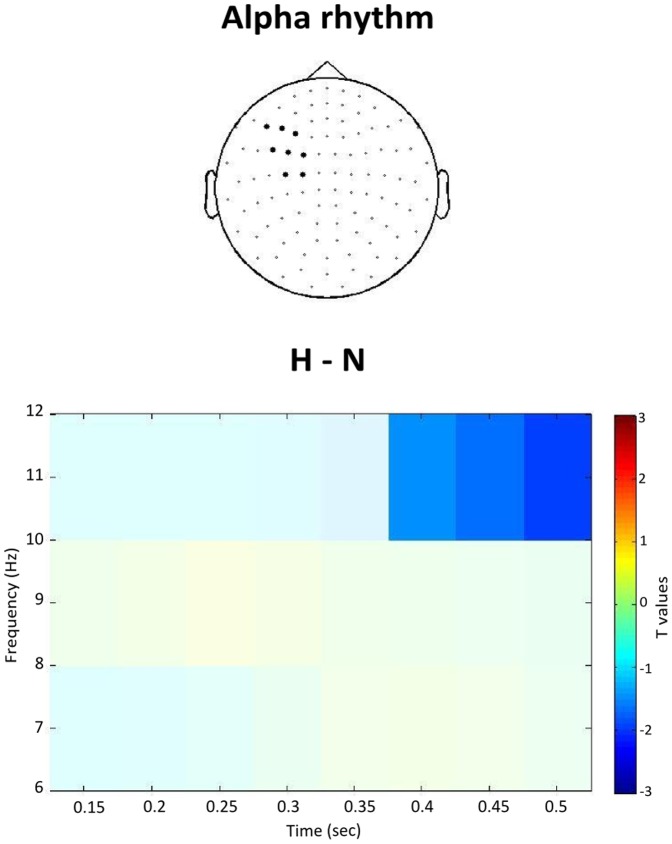
Statistical outcome for the alpha rhythm. T-values on a time-frequency map related to the H-N contrast, showing a significant cluster (saturated colours) on the average of the selected left-hemispheric hand-related sensors.

We compared beta and alpha power between each experimental condition (H, F) and the control condition N on those channels selected with the localizer task. Both the H and the F condition showed significantly stronger beta suppression than N after stimulus onset. Specifically, the H condition showed stronger beta modulation than N in the left hemisphere (*p* = .04; Fig. 3a), whereas no cluster was found in the right hemisphere. As shown in Fig. 4a, the oscillatory effect related to H verb processing became significant at around 200 msec post-stimulus onset. Similarly, the F-N contrast revealed significant beta modulation starting at around 200 msec post-stimulus onset on three right centrolateral channel pairs (*p* = .04; Fig. 3b and 4b), while no significant effect was observed on the left centrolateral sensors. While the H-N contrast showed an oscillatory modulation in the 20−24 Hz beta range, lower beta band modulation was observed in the F-N contrast (15–20 Hz). To confirm somatotopic distribution of beta modulation, we contrasted H and F conditions with N condition in the sensors selected for the non-corresponding extremity. No significant cluster emerged in either case (all *p*>.1). The alpha rhythm also showed significant suppression in the H-N contrast on left hemisphere hand-related channels (*p* = .03; Fig. 5). The oscillatory modulation occurred later compared to beta, namely at around 400 msec post word onset. No significant cluster emerged for the F-N contrast on foot-related channels (*p* = .46). Also in the alpha frequency range, the contrasts H-N and F-N on the sensors selected for the non-corresponding extremity provided no significant result (*p* = .34).

To determine the influence of imageability on oscillatory patterns of activation, we contrasted all high versus low imageability words independently from condition on the selected motor areas. No main effect of imageability on the selected motor regions was found during early lexico-semantic verb processing, as no significant cluster (*p* = .17) was found on the hand- and foot-related channels in the beta range. Besides, the analysis of possible interaction between imagery and condition resulted in no significant cluster for the H-N contrast (*p* = .18) and in no cluster for the F-N contrast. Similarly, no main effect of imagery and no interaction between condition and imagery were found for the alpha oscillations (all *p*>.1). To check whether the lack of significance was due to the halved number of trials in the high and low imagery condition, we tested the lexico-semantic effect on those same trials for the following contrasts: (a) the high imageable H-N and F-N contrasts and (b) the low imageable H-N and F-N contrasts. Indeed, the H-N contrast remained statistically significant both for the high (*p* = .007) and the low (*p* = .04) imageability subcondition, thus suggesting that the number of the trials was adequate. This was however not the case for the F-N contrast, which did not reach significance neither in the high nor in the low imageability condition (all *p*>.5).

## Discussion

The aim of the present study was to test the somatotopically distributed recruitment of cortical motor areas during action verb understanding in terms of modulations in the beta and alpha frequency ranges. The somatotopic information derived by a localizer task and the application of a cluster-based non-parametric statistical approach allowed us to find significant oscillatory effects accompanying the processing of single verbs. Specifically, we hypothesized that the processing of body-related verbs produces beta and alpha power suppression at around 200 msec post word onset in sensorimotor cortical areas that are engaged in the respective action execution. While we did observe power suppression in both frequency bands, different pattern emerged as for timing. As predicted, lexico-semantic processing of hand- and foot-related actions was accompanied by a stronger beta power suppression than the processing of non-body-related verbs on the cortical motor portion of hands and feet, respectively, around 200 msec. The H-N contrast revealed beta and alpha modulation in the left, but not in the right hemisphere. This asymmetric pattern of activation is in line with previous results showing left-lateralized power decrease during the reading of hand verbs [39] and covert verb generation [38]. Under the assumption that beta suppression represents neural activation [54], [55], [56], our findings also agree with those from fMRI studies showing left-lateralized neural activity during action-related language processing [11], [12], [14], [15], [16]. Consistently, inhibition of reaction times during the processing of hand action verbs was induced with TMS on the left, but not on the right hemispheric hand portion of the motor cortex [57]. Investigating the relation of lesion sites and behavioural performance on lexical and conceptual action processing, Kemmerer et al. [58] behaviourally tested 226 patients with cerebral lesions, from 147 of whom anatomical data were also obtained. Significant impairment of lexical and conceptual knowledge of actions was exclusively found in patients with left hemispheric lesions including hand-related motor areas. Although less prominently, the right hemisphere is also likely to play a role in verb processing, as shown in a study on patients with right frontal lobe lesions [59]. Beta suppression on bilateral mouth and hand regions was previously found during silent noun reading followed by delayed reading aloud, where suppression was further reinforced [60]. However, beta suppression in left-hemispheric cortical mouth areas started earlier and was stronger compared with the right hemisphere in fluent speakers. It is worth noting that while Salmelin et al. [60] addressed mental preparation for speech production as a possible explanation for the 20 Hz attenuation, the beta suppression found in the present study emerged in effector-related (hand and foot) motor areas and was stronger for H/F than N verbs. Furthermore, we did not apply a word generation task, thus minimizing the articulatory preparatory mechanisms related to overt speech in motor areas. Our results therefore point to a genuine difference between body-related and non-body-related verb processing and provide additional evidence for a prevalent role of the left cortical motor areas in processing action words.

In the studies mentioned above, all participants (and the large majority in Kemmerer et al.’s study [58]) were right-handed. Given that the processing of uni-manual action words is biased towards the dominant hand [61], left-lateralized brain activation in right-handed subjects, as found in the present study, is no great surprise. Indeed, the well-known left-biased asymmetry related to language processing seems to depend on handedness, as shown by an almost linear relationship between the degree of handedness and the direction of language dominance in terms of word generation in 326 healthy individuals [62]. Moreover, co-lateralization of praxis and language networks was demonstrated in individuals with right and with left language dominance [63]. In this context, it is of interest that lateralized beta power suppression may serve as an indicator of the side of language lateralization as well [64], [38].

The use of non-body-related verbs in our paradigm permitted us to gain a view of neural activations subtending abstract words. As shown in Fig. 3a, the processing of abstract (N) verbs was also accompanied by beta suppression on the hand-related motor area, although this was significantly less when compared to the H condition. This finding agrees with the claim that abstract words are also embodied in perception and action. Specifically, Barsalou [65] proposed that abstract concepts are grounded in complex simulations of combined physical and introspective events that convey sensorimotor details. Vigliocco et al. [66] interpreted the apparent dichotomy between concrete and abstract word meanings as a preponderance of sensorimotor information, which is more abundant in concrete than abstract words. The hypothesized embodiment of abstract concepts is supported by neuroscientific studies. Using a similar paradigm to ours, Rüschemeyer et al. [15] found sensorimotor blood-oxygenation-level-dependent (BOLD) activation both for concrete and abstract verbs, although less prominently for the latter. Similar results were observed for the comprehension of concrete and abstract sentences [67]. Both metaphoric/idiomatic and literal action sentences were shown to activate regions associated with sensorimotor processing [12], [68], [69]. Glenberg et al. [70] showed that task-related modulation of the motor system by means of manually transferring items towards or away from the body affected the comprehension of abstract as well as concrete sentences referring to transfer. Altogether, these findings point to a recruitment of motor cortical areas also for the processing of abstract words. Interestingly, implicit processing of ortho-phonological statistical regularities also activated the motor area, as shown in the fMRI study of Zubicaray et al. [71]. The authors found that non-words containing endings with probabilistic cues predictive of verb status, evoked enhanced activity compared with non-words with endings predictive of noun status, in a similar motor area as the one activated for action verbs. It might be reasoned that beta suppression shown by abstract verbs in motor areas in the present study partly depends on the typical verb ending. However, this is not the case because the hand and the non-body conditions showed a statistical difference that can not be explained by the typical verb ending.

Some differences between the H and the F condition emerged in the beta range, as shown by the respective contrasts with the control condition (Fig. 4). First, hand and foot verbs modulated beta oscillations in slightly different frequency bands. As suggested by Pfurtscheller et al. [72], each primary sensorimotor area may have its own intrinsic rhythm. Also, the corticomuscular analysis conducted for localization purposes showed on average highest coherence in the 20–24 Hz beta range both for hands and feet contraction (data not shown), which is in agreement with previous reports [73]. This beta band is identical to the one showing an effect during linguistic processing of hand verbs. In our data, foot contraction and foot word processing apparently do not share the same beta frequency band. This difference possibly arises due to the fact that coherence measures (corticomuscular coupling) and power measures are not identical. Alternatively, it is possible that beta oscillations exhibit task-specificity and do not completely overlap between an isometric contraction and a linguistic task. However, since the time window of effect is comparable for the H-N and F-N contrast, both processes are likely to share the same function. Second, while beta suppression emerged on the left hemisphere in the H-N contrast, it was slightly right-lateralized in the F-N contrast. In our opinion, the foot-related lateralization results should be interpreted with caution. As the foot motor representation is to some extent buried in the interhemispheric fissure, it is difficult to accurately localize its activation by means of MEG. This is confirmed by the large overlap between sensors showing activation during right and left foot contraction (Fig. 2) and might also explain why the F-N contrast did not reach significance neither in the high nor in the low imageability condition.

The pattern of beta decrease found in the present study is in line with previous investigations on verb generation [38] and silent sentence reading [31] as regards timing and hemispheric lateralization, respectively. Although in the study of van Elk et al. [31] the beta suppression during action verb processing reached significance at 400–600 ms after word onset, it was visibly present as early as 200 ms. It should also be noted that the task applied in our study required neither semantic processing nor awareness of the stimuli’s body-relatedness. Our results therefore imply that even lower linguistic processing levels than the semantic one may engage motor brain regions, thus corroborating previous findings [42]. An interesting issue which remains to be addressed is whether the depth of cognitive action processing modulates the power of beta oscillations on motor regions.

Like beta, the alpha rhythm was also modulated by the body-relatedness of verbs, as hand-related verbs showed significantly stronger alpha suppression than non-body verbs. This finding replicates that of van Elk et al. [31] at single verb level, although at a longer latency, namely at 400 instead of 200 msec post-stimulus onset. In contrast to beta oscillations, which are thought to largely reflect activity of the motor cortex, the 10 Hz signal was suggested to have a somatosensory origin [74]. It is therefore possible that reading body-related verbs also elicited a somatosensory component beyond the motor one associated to beta oscillations. In a similar manner to an executed movement, the processing of an action verb may be also sequenced into earlier processing steps, i.e. motor command associated with beta modulation and a later processing paralleling sensory feedback associated with alpha rhythm. This assumption would further expand the embodiment framework into the temporal domain, which should be focussed on in later studies. However, the processing of foot-related verbs did not result in alpha modulation. Possibly, the hand area is in closer contact with language as language has been suggested to evolve from manual gesture [9]. An alternative hypothesis on the functional role of alpha is that alpha reflects later semantic processes that dissociate from somatotopic language-related aspects. This might explain the absence of alpha modulation in the foot region.

As abstract verbs were less imageable than concrete verbs, we tested whether the level of imageability corresponded to significant oscillatory modulation and whether imagery processes played a role in the oscillatory effect found in the H-N and F-N contrast. The results showed similar oscillatory correlates for high and low imageable verbs and no interaction between imageability and condition on the selected hand and foot motor areas. Imageability appeared to play no role in the time-window between 150 and 500 msec post-stimulus onset. One noteworthy aspect is that a later onset of oscillatory modulations related to motor imagery processing has been reported previously [25], [26], [27]. Altogether, these findings rule out the hypothesis that imagery processes might have caused or modulated the oscillatory activation during lexico-semantic processing.

One limitation of the present study is that the match of the stimuli across conditions resulted in higher database frequency of non-body compared to body-related words. However, high-frequency words were shown to elicit a larger beta power suppression than low-frequency words [75]. If frequency had affected our results, we should have found larger beta suppression for the N than for the H/F condition. Alternatively, the higher frequency of abstract words might have hidden a power difference between the experimental and control conditions. In fact, our results show that both experimental conditions induced larger beta suppression than the control condition. It is therefore unlikely that this oscillatory modulation depends on differences in lexical frequency between conditions.

To summarise, we tested the grounded cognition framework on brain oscillatory activity and showed for the first time that silent reading of action words in a lexical decision task elicited significant beta power suppression in a similar fashion to limb movements and according to a somatotopic distribution. The differential engagement of motor areas in body-related versus abstract verb processing was time-specific, as it was observed between 200 and 250 msec after word onset. Moreover, a possible somatosensory processing accompanying hand-related verb reading was suggested by significant power suppression in the alpha frequency range at later latencies. The present study lays the groundwork for an investigation of interaction and coherence between different brain areas that are involved, possibly essentially, in the neurobiology of language.

## Supporting Information

Table S1 **Stimuli used in the three conditions and relative indexes of familiarity (Fam.), imageability (Imag.), frequency (Freq.), and length (Lgth.).** Means and standard deviations of various parameters are shown for each condition.
(DOC)
