# Correction: Grasping Hand Verbs: Oscillatory Beta and Alpha Correlates of Action-Word Processing

**DOI:** 10.1371/journal.pone.0161985

**Published:** 2016-08-24

**Authors:** Valentina Niccolai, Anne Klepp, Hannah Weissler, Nienke Hoogenboom, Alfons Schnitzler, Katja Biermann-Ruben

A reference is omitted from the third sentence of the second paragraph of the Introduction.

The sentence should read: Recently, our research group showed by means of magnetoencephalography (MEG) somatotopic activation of motor areas accompanying the processing of visually presented single verbs (Klepp et al,. 2014).

The reference is: Klepp A, Weissler H, Niccolai V, Terhalle A, Geisler H, Schnitzler A, Biermann-Ruben K (2014) Neuromagnetic hand and foot motor source recruited during action verb processing. Brain Lang 128(1): 41–52. doi: 10.1016/j.bandl.2013.12.001.
