# Supplementary material for: Grasping Hand Verbs: Oscillatory Beta and Alpha Correlates of Action-Word Processing
Source: PLoS One. 2014 Sep 23;9(9):e108059. doi: 10.1371/journal.pone.0108059 (PMC4172661; doi:10.1371/journal.pone.0108059)
Supplement: Table S1 — Stimuli used in the three conditions and relative indexes of familiarity (Fam.), imageability (Imag.), frequency (Freq.), and length (Lgth.). Means and standard deviations of various parameters are shown for each condition. (DOC) [file pone.0108059.s001.doc]

| **1.Hand verbs** | | | | | | **2. Foot verbs** | | | | | | **3. Non-body verbs** | | | | | |
| --- | --- | --- | --- | --- | --- | --- | --- | --- | --- | --- | --- | --- | --- | --- | --- | --- | --- |
|  | Fam. | Imag. | Freq. | Lgth | English |  | Fam. | Imag. | Freq. | Lgth | English |  | Fam. | Imag. | Freq. | Lgth | English |
| **angeln** | 3.37 | 3.50 | 15 | 6 | to fish | **eilen** | 3.37 | 3.33 | 14 | 5 | to hurry | **achten** | 3.33 | 2.07 | 10 | 6 | to respect |
| **basteln** | 3.67 | 3.60 | 13 | 7 | to tinker | **fliehen** | 3.60 | 3.37 | 12 | 7 | to flee | **ähneln** | 3.47 | 2.03 | 13 | 6 | to resemble |
| **binden** | 3.47 | 3.50 | 12 | 6 | to tie | **flitzen** | 3.10 | 3.21 | 15 | 7 | to dash | **ahnen** | 3.27 | 2.20 | 12 | 5 | to suspect |
| **boxen** | 3.40 | 3.47 | 12 | 5 | to box | **flüchten** | 3.63 | 3.20 | 12 | 8 | to escape | **bangen** | 2.70 | 2.20 | 12 | 6 | to fear |
| **buddeln** | 3.07 | 3.40 | 16 | 7 | to dig | **folgen** | 3.70 | 3.40 | 9 | 6 | to follow | **bessern** | 3.17 | 1.93 | 14 | 7 | to improve |
| **falten** | 3.50 | 3.73 | 15 | 6 | to fold | **gehen** | 3.97 | 3.93 | 6 | 5 | to walk | **büffeln** | 3.00 | 2.86 | 16 | 7 | to swot |
| **fassen** | 3.57 | 3.43 | 11 | 6 | to grab | **grätschen** | 2.57 | 2.80 | 17 | 9 | to straddle | **bürgen** | 2.67 | 1.80 | 16 | 6 | to vouch |
| **feilen** | 3.17 | 3.47 | 14 | 6 | to file | **hasten** | 2.77 | 2.87 | 16 | 6 | to rush | **büßen** | 2.73 | 1.70 | 14 | 5 | to atone |
| **flechten** | 3.00 | 3.50 | 16 | 8 | to plait | **hinken** | 3.10 | 3.40 | 15 | 6 | to limp | **denken** | 3.93 | 2.47 | 9 | 6 | to think |
| **fuchteln** | 2.76 | 3.10 | 18 | 8 | to wave about | **hocken** | 3.40 | 3.53 | 14 | 6 | to squat | **dulden** | 3.10 | 2.23 | 13 | 6 | to tolerate |
| **greifen** | 3.90 | 3.90 | 10 | 7 | to grasp | **hoppeln** | 2.77 | 2.93 | 17 | 7 | to lollop | **ehren** | 3.20 | 2.27 | 13 | 5 | to honor |
| **häkeln** | 2.97 | 3.33 | 19 | 6 | to crochet | **hopsen** | 2.72 | 3.33 | 17 | 6 | to skip | **eignen** | 3.17 | 1.73 | 13 | 6 | to suit |
| **kehren** | 3.13 | 3.40 | 12 | 6 | to sweep | **humpeln** | 3.23 | 3.37 | 17 | 7 | to hobble | **folgern** | 3.47 | 2.10 | 15 | 7 | to conclude |
| **klatschen** | 3.77 | 3.77 | 13 | 9 | to clap | **hüpfen** | 3.63 | 3.77 | 14 | 6 | to hop | **fügen** | 2.80 | 1.67 | 13 | 5 | to comply |
| **kneifen** | 3.47 | 3.57 | 16 | 7 | to pinch | **joggen** | 3.83 | 3.87 | 15 | 6 | to jog | **glauben** | 3.90 | 2.17 | 9 | 7 | to believe |
| **kneten** | 3.47 | 3.70 | 17 | 6 | to knead | **kicken** | 2.97 | 3.33 | 14 | 6 | to kick | **gönnen** | 3.43 | 2.10 | 12 | 6 | to grant |
| **knoten** | 3.23 | 3.57 | 19 | 6 | to knot | **knien** | 3.40 | 3.72 | 16 | 5 | to kneel | **grämen** | 2.03 | 1.87 | 16 | 6 | to grieve |
| **melken** | 3.20 | 3.37 | 16 | 6 | to milk | **latschen** | 2.80 | 3.13 | 18 | 8 | to traipse | **grübeln** | 3.37 | 2.50 | 15 | 7 | to brood |
| **nähen** | 3.47 | 3.70 | 15 | 5 | to sew | **laufen** | 4.00 | 3.90 | 8 | 6 | to run | **hadern** | 2.47 | 1.83 | 15 | 6 | to quarrel with |
| **paddeln** | 3.10 | 3.60 | 16 | 7 | to paddle | **radeln** | 3.57 | 3.70 | 15 | 6 | to cycle | **hassen** | 3.77 | 2.47 | 14 | 6 | to hate |
| **pellen** | 2.90 | 3.30 | 18 | 6 | to peel | **rasen** | 3.50 | 3.24 | 13 | 5 | to rush | **herrschen** | 3.37 | 2.57 | 12 | 9 | to govern |
| **pflücken** | 3.30 | 3.77 | 15 | 8 | to pick | **rennen** | 3.90 | 4.00 | 12 | 6 | to run | **hoffen** | 3.87 | 2.20 | 9 | 6 | to hope |
| **rubbeln** | 3.10 | 3.43 | 18 | 7 | to scour | **scharren** | 2.40 | 2.93 | 16 | 8 | to scrabble | **irren** | 3.37 | 2.17 | 14 | 5 | to err |
| **rütteln** | 3.27 | 3.37 | 13 | 7 | to shake | **schleichen** | 3.53 | 3.57 | 14 | 10 | to creep | **meinen** | 3.87 | 2.17 | 8 | 6 | to mean |
| **schälen** | 3.60 | 3.60 | 15 | 7 | to peel | **schlendern** | 3.13 | 3.23 | 14 | 10 | to saunter | **meistern** | 3.13 | 1.80 | 12 | 8 | to master |
| **scheuern** | 2.90 | 3.33 | 18 | 8 | to rub | **schlittern** | 2.73 | 3.27 | 16 | 10 | to slither | **merken** | 3.90 | 2.20 | 11 | 6 | to notice |
| **schlagen** | 3.73 | 3.77 | 10 | 8 | to beat | **schlurfen** | 2.80 | 3.20 | 17 | 9 | to scuffle | **mogeln** | 3.27 | 2.30 | 17 | 6 | to cheat |
| **schleifen** | 3.17 | 3.20 | 14 | 9 | to whet | **schreiten** | 2.90 | 2.87 | 14 | 9 | to stride | **mögen** | 3.97 | 2.41 | 10 | 5 | to like |
| **schleudern** | 3.23 | 3.23 | 15 | 10 | to hurl | **skaten** | 2.97 | 3.47 | 17 | 6 | to skate | **plagen** | 2.93 | 2.10 | 13 | 6 | to afflict |
| **schneidern** | 3.10 | 3.20 | 16 | 10 | to tailor | **springen** | 3.97 | 3.90 | 11 | 8 | to jump | **planen** | 3.83 | 2.47 | 10 | 6 | to plan |
| **schnipsen** | 3.03 | 3.70 | 19 | 9 | to flick | **sprinten** | 3.30 | 3.60 | 16 | 8 | to sprint | **raten** | 3.73 | 2.31 | 11 | 5 | to guess |
| **schnitzen** | 3.17 | 3.50 | 17 | 9 | to carve | **spurten** | 2.67 | 2.97 | 17 | 7 | to spurt | **schätzen** | 3.70 | 2.17 | 10 | 8 | to estimate |
| **schnüren** | 3.17 | 3.17 | 14 | 8 | to lace | **stampfen** | 3.10 | 3.30 | 15 | 8 | to stomp | **schulden** | 3.37 | 1.90 | 15 | 8 | to owe |
| **schreiben** | 4.00 | 4.00 | 9 | 9 | to write | **stapfen** | 2.93 | 3.00 | 16 | 7 | to trudge | **schummeln** | 3.27 | 2.50 | 17 | 9 | to cheat |
| **schrubben** | 3.13 | 3.47 | 17 | 9 | to scrub | **stehen** | 3.93 | 3.90 | 6 | 6 | to stand | **sehnen** | 3.07 | 2.23 | 14 | 6 | to yearn |
| **spitzen** | 2.83 | 2.97 | 14 | 7 | to sharpen | **steigen** | 3.60 | 3.33 | 8 | 7 | to climb | **sinnen** | 2.17 | 1.70 | 17 | 6 | to ponder |
| **stapeln** | 3.43 | 3.60 | 13 | 7 | to pile | **steppen** | 2.47 | 2.73 | 17 | 7 | to tap-dance | **streben** | 3.27 | 2.23 | 12 | 7 | to aspire |
| **stochern** | 2.90 | 3.13 | 17 | 8 | to stoke | **stolpern** | 3.60 | 3.67 | 14 | 8 | to stumble | **täuschen** | 3.50 | 2.20 | 13 | 8 | to fool |
| **stopfen** | 3.27 | 2.90 | 13 | 7 | to stuff | **strampeln** | 3.07 | 3.40 | 16 | 9 | to struggle | **trauen** | 3.53 | 2.20 | 11 | 6 | to trust |
| **stricken** | 3.33 | 3.40 | 15 | 8 | to knit | **stürmen** | 3.23 | 2.93 | 12 | 7 | to storm | **träumen** | 3.80 | 3.00 | 11 | 7 | to dream |
| **stupsen** | 3.07 | 3.27 | 19 | 7 | to nudge | **tänzeln** | 2.83 | 3.03 | 17 | 7 | to prance | **trotzen** | 2.90 | 2.10 | 14 | 7 | to defy |
| **tippen** | 3.50 | 3.40 | 14 | 6 | to tap | **torkeln** | 2.70 | 3.43 | 17 | 7 | to stagger | **wagen** | 3.37 | 2.00 | 11 | 5 | to dare |
| **trommeln** | 3.43 | 3.69 | 15 | 8 | to drum | **trampeln** | 3.17 | 3.47 | 16 | 8 | to trample | **werten** | 3.20 | 1.90 | 12 | 6 | to assess |
| **wedeln** | 2.70 | 3.07 | 16 | 6 | to waggle | **treten** | 3.87 | 3.77 | 9 | 6 | to kick | **wissen** | 3.90 | 2.27 | 7 | 6 | to know |
| **wickeln** | 3.27 | 3.37 | 15 | 7 | to wrap | **trotten** | 2.47 | 2.87 | 16 | 7 | to trot | **wundern** | 3.80 | 2.43 | 12 | 7 | to marvel |
| **winken** | 3.63 | 3.87 | 13 | 6 | to wave | **wandern** | 3.73 | 3.90 | 12 | 7 | to hike | **wünschen** | 3.90 | 2.30 | 10 | 8 | to wish |
| **zerren** | 3.00 | 3.33 | 14 | 6 | to drag | **watscheln** | 2.47 | 2.87 | 17 | 9 | to waddle | **zaudern** | 1.97 | 1.77 | 17 | 7 | to tarry |
| **zupfen** | 3.10 | 3.07 | 16 | 6 | to pluck | **wippen** | 3.00 | 3.23 | 16 | 6 | to seesaw | **zweifeln** | 3.77 | 2.47 | 12 | 8 | to doubt |
| **MEAN (±SD)** | 3.27  ± 0.29 | 3.45  ± 0.25 | 14.94  ± 2.45 | 7.15  ± 1.25 |  | **MEAN (±SD)** | 3.21  ± 0.47 | 3.36  ± 0.35 | 14.21  ± 3.05 | 7.08  ± 1.35 |  | **MEAN**  **(±SD)** | 3.31  ± 0.50 | 2.17  ± 0.29 | 12.63  ± 2.48 | 6.42  ± 1.05 |  |
